# Optogenetic stimulation promotes Schwann cell proliferation, differentiation, and myelination *in vitro*

**DOI:** 10.1038/s41598-019-40173-w

**Published:** 2019-03-05

**Authors:** Kyuhwan Jung, Ji Hye Park, Sung-Yon Kim, Noo Li Jeon, Sung-Rae Cho, Sujin Hyung

**Affiliations:** 1 Graduate Program of Nano Science and Technology, Graduate School of Yonsei University, Seoul, Korea; 2 Gradaute Program of Translational Neuroscience, Institute for Clinical Neurobiology, University of Wuerzburg, Wuerzburg, Germany; 3 Department of Biophysics and Chemical Biology, Seoul National University, Seoul, South Korea; 4 Multiscale Mechanical Design School of Mechanical and Aerospace Engineering Institute of Advanced Machinery and Design, Seoul National University, Seoul, Korea; 5 Institute of Bioengineering, Seoul National University, Seoul, Korea; 6 Department and Research Institute of Rehabilitation Medicine, Yonsei University College of Medicine, Seoul, Korea; 7 BK21 Plus Transformative Training Program for Creative Mechanical and Aerospace Engineers, Seoul National University, Seoul, Korea; 8 Present Address: Department of Molecular Biology and Genetics, Weill Institute for Cell and Molecular Biology, Cornell University, Ithaca, USA

## Abstract

Schwann cells (SCs) constitute a crucial element of the peripheral nervous system, by structurally supporting the formation of myelin and conveying vital trophic factors to the nervous system. However, the functions of SCs in developmental and regenerative stages remain unclear. Here, we investigated how optogenetic stimulation (OS) of SCs regulates their development. In SC monoculture, OS substantially enhanced SC proliferation and the number of BrdU^+^-S100ß^+^-SCs over time. In addition, OS also markedly promoted the expression of both Krox20 and myelin basic protein (MBP) in SC culture medium containing dBcAMP/NRG1, which induced differentiation. We found that the effects of OS are dependent on the intracellular Ca^2+^ level. OS induces elevated intracellular Ca^2+^ levels through the T-type voltage-gated calcium channel (VGCC) and mobilization of Ca^2+^ from both inositol 1,4,5-trisphosphate (IP_3_)-sensitive stores and caffeine/ryanodine-sensitive stores. Furthermore, we confirmed that OS significantly increased expression levels of both Krox20 and MBP in SC-motor neuron (MN) coculture, which was notably prevented by pharmacological intervention with Ca^2+^. Taken together, our results demonstrate that OS of SCs increases the intracellular Ca^2+^ level and can regulate proliferation, differentiation, and myelination, suggesting that OS of SCs may offer a new approach to the treatment of neurodegenerative disorders.

## Introduction

Axon regeneration and remyelination after injury are limited in the central nervous system (CNS) of adult mammals, but recovery capacity is considerably greater in the peripheral nervous system (PNS). Schwann cells (SCs), a glial cell type of the PNS, are crucial for regeneration in the PNS. After peripheral nerve injury, SCs immediately transform into a dedifferentiated state and activate proliferation *per se*, forming bands of Büngner in which SCs release numerous neurotrophic factors that guide axonal extensions and induce infiltration of macrophages into the injury site, to in turn remove cell debris^1–5^. Dedifferentiated SCs can undergo redifferentiation through upregulation of myelin-associated genes and remyelinate the regenerating axons for complete functional recovery, but regeneration of the PNS with an injured area more than 3 cm in size remains relatively unexplored^6–8^. The potential efficacy of SCs is dependent on patient age, regeneration time, and injury extent^9^. Intensive treatment strategies have been shown to promote axon regeneration, but the molecular mechanisms underlying the regenerative response and long-distance axon regeneration are not fully understood. More advanced practical methods for determining the roles of SCs in promoting axon regeneration are essential.

Optogenetics has gained attention as a promising biological technology, in which genetically modified cells within complex neural tissues are regulated using light of a specific wavelength after introducing light-sensitive microbial opsins, such as channelrhodopsin-2 (ChR-2), halorhodopsin (NpHR), and archaerhodopsin^10–13^. This revolutionary technique can control the electrical processes of specific types of cells, including cell signaling, modulation, etc.; in particular, it affords unprecedented control of neural activity with high spatiotemporal precision in many types of nervous system investigations. To date, numerous optogenetic studies have demonstrated modulation of the neural system at the molecular and cellular levels through neuronal excitation^12,14–16^, inhibition^17–21^, and biochemical control^22–26^ of intracellular membranes, the endoplasmic reticulum, nuclear complexes, and mitochondria. More recently, a few studies on optogenetic applications have focused on glial cells, such as astrocytes, in the CNS^27–31^. Regarding optogenetically manipulated astrocytes, their activation can be separated from that of nearby neurons, which may shed light on the role of glial cells in highly complex brain functions including modulation of synaptic transmission^27,31^, regulation of response selectivity of visual cortex neurons^29,30^, control of the sleep-wake cycle in the posterior hypothalamus^28^, and activation of ATP release^27^. In contrast to astrocytes, the possibility of optogenetic SCs has not been reported. When optogenetic-mediated dorsal root ganglia cells are stimulated, SCs have been shown to respond to nerve stimuli, and the proliferation and migration of SCs were regulated by optogenetic neuronal stimulation^32^. Accumulating evidence has shown that SCs play essential roles in neuronal survival^33,34^, axon growth^35,36^, and myelination^37,38^, but it has proven difficult to investigate their role in neuron-glia complexes. Although the actions of SCs are crucial to neurons, fundamental questions regarding their significance to the PNS remain unresolved.

Here, we investigated an optogenetic approach that serves as a useful extension of the tools currently available to study SC function in the PNS. We focused on how optogenetic stimulation (OS) of SCs affects SC development, such as proliferation, differentiation, and myelination, and demonstrated that OS of SCs not only induces SC proliferation but also promotes the differentiation and myelination of SCs in SC monoculture. Moreover, we provided evidence that OS activity is correlated with a change in the intracellular Ca^2+^ level. We discuss below how OS affects the differentiation and myelination of SCs in a coculture model of SC and motor neuron (MN) cells.

## Results

### OS induces SC proliferation

**Figure 1:**
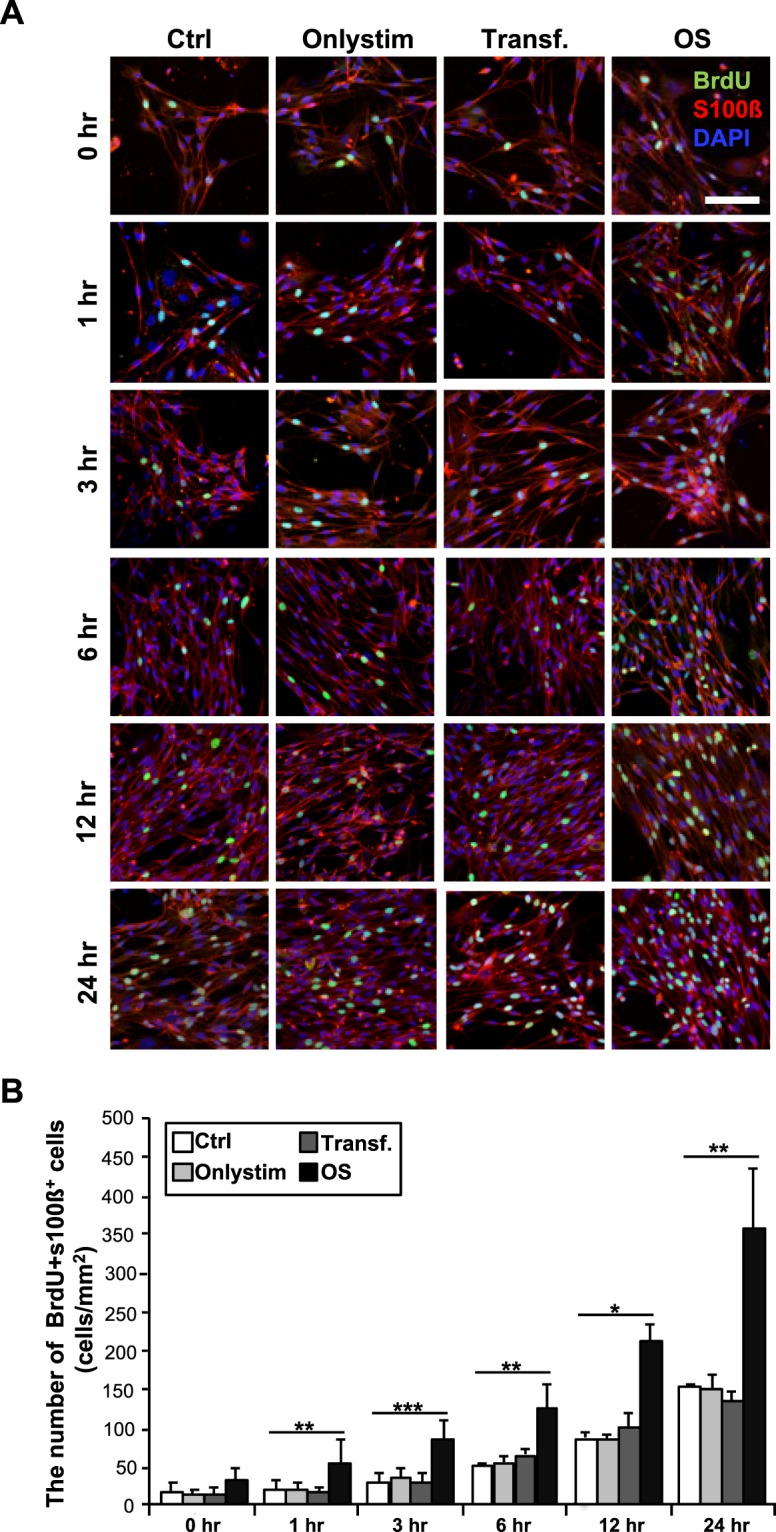
Increase in the number of BrdU^+^-S100ß^+^-Schwann cells (SCs) in SC monoculture with optogenetic stimulation (OS). SCs were cultured on poly-L-lysine (PLL)-coated coverslips under four different conditions: non-transfected without light-emitting diode (LED) stimulation (Ctrl), non-transfected with LED stimulation (Onlystim), transfected without LED stimulation (Transf.), and transfected with LED stimulation (OS), and SCs from each treatment were fixed at 0, 1, 3, 6, 12, and 24 h, as indicated. (**A**) Representative images and (**B**) quantification of SCs stained with 5-bromo-2′-deoxyuridine (BrdU; green), which is a marker of proliferating cells, S100ß (red), a specific SC cytoplasm marker, and 4′,6-diamidino-2-phenylindole (DAPI; blue) are shown. Note the marked elevation of BrdU^+^-S100ß^+^-SCs with OS. Graph shows means ± standard error of the mean (SEM) from five independent experiments (unpaired two-tailed-*t* test with Welch’s correction). Scale bar, 50 µm. *p = 3.8 × 10^−2^ (0 h); **p = 8.1 × 10^−3^ (1 h); ***p = 3.0 × 10^−4^ (3 h); **p = 3.4 × 10^−3^(6 h); *p = 3.7 × 10^−2^ (12 h); **p = 8.3 × 10^−3^ (24 h).

To investigate whether optogenetic-mediated SCs could perform the general biological functions of SCs, including proliferation and differentiation, we first performed SC proliferation analysis. SCs cultured on coverslips that had been pre-coated with poly-l-lysine (PLL) were transfected with 5 µg of calcium-translocating channelrhodopsin (CatCh) DNA. Before examining SC proliferation, we verified the efficiency of CatCh transfection in the SC monoculture with a YFP-expressing plasmid, which was strongly expressed in the majority of SCs at DIV 7, with 92.4 ± 2.6% of SCs being transfected (Suppl. Fig. S1). Next, we examined whether OS affected the number of SCs. To more accurately measure SC proliferation in the SC monoculture, we evaluated SC proliferation using 5-bromo-2′-deoxyuridine (BrdU), a thymidine analog that is incorporated into the DNA of proliferating SCs when they are in the DNA replication phase (i.e., S-phase). Furthermore, SCs were cultured under four different conditions: non-transfected SCs without light emitting diode (LED) stimulation (Ctrl), non-transfected SCs with LED stimulation (Onlystim), transfected SCs without LED stimulation (Transf.), and transfected SCs with LED stimulation (OS), and were analyzed at 0, 1, 3, 6, 12, and 24 h after the LED irradiator was applied. Through measurement of the number of SCs stained with BrdU, the specific SC cytoplasm marker S100β, and 4′,6-diamidino-2-phenylindole (DAPI), we found that transfected SCs proliferated rapidly with OS after only 1 h (Fig. 1A,B). In addition, BrdU expression increased markedly with OS of SCs compared to other conditions after 1 h, and there were no significant differences among the Ctrl, Onlystim, and Transf. groups. The number of BrdU^+^-S100ß^+^-SCs increased by about 2.6-, 2.7-, 2.4-, 2.4-, and 2.3-fold at 1, 3, 6, 12, and 24 h, respectively (Fig. 1B). These results show that OS of SCs activates SC proliferation.

### OS promotes the expression of myelin-associated proteins in SC monoculture

**Figure 2:**
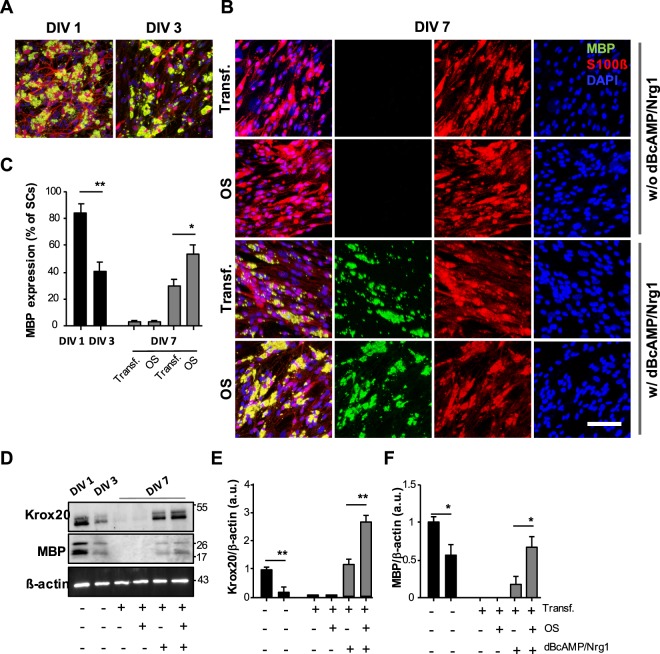
Elevation of the expression levels of myelin-associated proteins with OS in SC monoculture. SCs were grown in culture medium in the presence or absence of a mixture of dBcAMP (1 mM) and NGR1 (10 nM) with or without OS. Transfected SCs were analyzed at DIV 1, 3, and 7 by immunostaining with antibodies against myelin basic protein (MBP; green), S100ß (red), and DAPI (blue,) as well as western blotting. (**A**,**B**) Representative confocal images and (**C**) quantification of MBP^+^-SCs are shown. Note the increase in MBP-expressing SCs on treatment with a mixture of dBcAMP/NGR1 and OS at DIV 7. Graph shows means ± SEM from three independent experiments (unpaired two-tailed-*t* test with Welch’s correction). Scale bar, 50 µm. **p = 2.97 × 10^−3^ and *p = 2.01 × 10^−2^. (**D**–**F**) The levels of Krox20 and MBP were determined through western blot analysis. (**D**) Representative immunoblots and (**E**,**F**) quantification of Krox20 and MBP protein levels are shown. Both Krox20 and MBP expression levels of transfected SCs treated with dBcAMP/NRG1 were enhanced with OS, with expression levels of Krox20 and MBP showing 2- and 4.8-fold increases compared to those without OS, respectively. Protein levels were normalized against the level of ß-actin, which was used as a loading control. Graph shows means ± SEM from four independent experiments (unpaired two-tailed-*t* test with Welch’s correction). **p = 4.98 × 10^−3^ (DIV 1 vs. DIV 3 of Krox20); **, *p* = 2.07 × 10^−3^ (Transf. vs. OS of Krox20 at DIV 7); *p = 1.15 × 10^−2^ (DIV 1 vs. DIV 3 of MBP); *p = 1.12 × 10^−2^ (Transf. vs. OS of MBP at DIV 7).

To investigate whether OS of SCs also affected the differentiation and myelination of SCs in monoculture, we analyzed the expression levels of myelin-associated proteins such as Krox20, which is an SC transcription regulatory factor driving the transition from non-myelinated to myelinated status, and myelin basic protein (MBP), which is a critical component of myelin sheaths. In a traditional SC culture environment, SCs without axonal contact progressively downregulate the expression of myelin-associated genes. Consistent with previous reports, we confirmed that SCs rapidly decrease their expression of myelin-associated proteins, and that the number of MBP^+^-SCs was substantially reduced at DIV 3. Thereafter, MBP expression became nearly undetectable after DIV 7 (Fig. 2A,C). OS of SCs did not affect MBP expression in this manner, and there was no significant difference between Transf. and OS under traditional culture conditions (Fig. 2B,C).

Recently, several papers have reported that SCs can be induced to differentiate into a myelinating phenotype through treatment with a high concentration of dibutyryl cAMP (dBcAMP, 1 mM) and neuregulin1 (NRG1, 10 nM) in an axon-free culture environment^39,40^. To further examine the effect of OS of SCs in SC culture medium treated with dBcAMP/NRG1, we added a high concentration of dBcAMP/NRG1 to the SC culture medium at DIV 3. SCs gradually increased their MBP expression in SC culture medium including dBcAMP/NRG1 at DIV 7 and, surprisingly, MBP expression was substantially enhanced by OS of SCs (Fig. 2B,C). When cultured in differentiation medium containing dBcAMP/NRG1, we observed that OS of SCs promoted SC differentiation and myelination, with 54.53 ± 6.32% of SCs being differentiated. In particular, MBP^+^-SCs exhibited an approximately 1.8-fold increase compared to the treatment without LED stimulation (Fig. 2C). Furthermore, we confirmed the levels of Krox20 and MBP expression using western blotting analysis. The expression levels of both Krox20 and MBP decreased progressively over time in the transitional SC culture medium, and there was no statistically significant difference between Transf. and OS at DIV 7 (Fig. 2D–F). However, when treated with dBcAMP/NRG1 in SC culture medium, the levels of Krox20 and MBP increased. Importantly, we found that the expression levels of Krox20 and MBP were strongly enhanced by OS of SCs, with Krox20 and MBP expression levels being increased by about 2- and 4.8-fold, respectively, compared to those without LED stimulation (Fig. 2E,F). Taken together, our results suggest that OS of SCs not only induces SC proliferation but also promotes SC differentiation and myelination, suggesting that OS of SCs might play an essential role as a gliomodulator.

### OS of SCs induces intracellular Ca^2+^ increase

**Figure 3:**
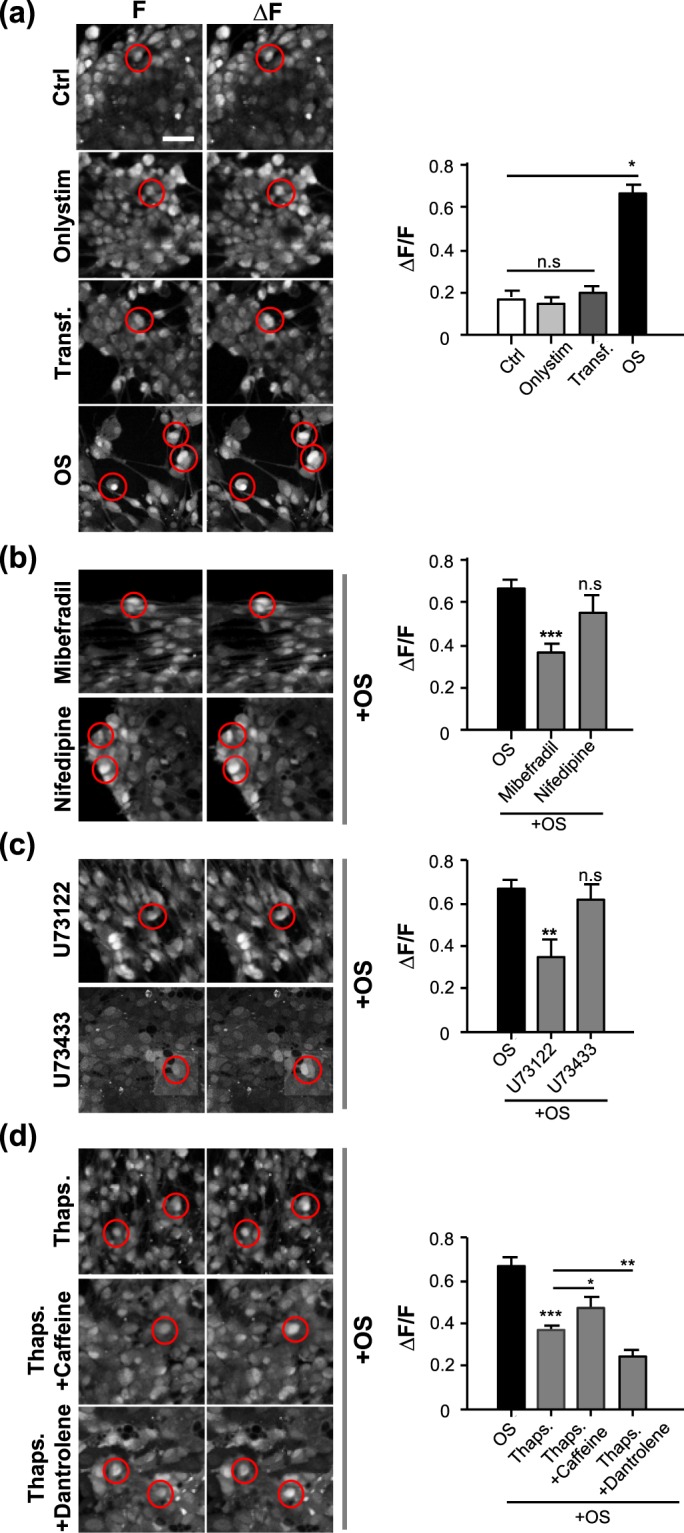
High intracellular Ca^2+^ triggered by OS in transfected SCs. Intracellular Ca^2+^ levels of SCs transfected with or without CatCh at DIV 3 were analyzed at DIV 4 using the Oregon Green^TM^ 488 BAPTA-1 AM staining kit. (**a**) Representative confocal images and quantification of four different conditions (Ctrl, Onlystim, Transf. and OS) are shown. Transfected SCs were treated with (**b**) mibefradil (40 nM, a T-type voltage-gated calcium channel [VGCC] blocker), nifedipine (20 nM, an L-type VGCC blocker), (**c**) U73122 (500 nM, a specific inhibitor of phospholipase C beta [PLC-ß]), U73433 (500 nM, a partially inactive structural analog of U73122), (**d**) Thaps. (thapsigargin, 200 nM; depletes1,4,5-trisphosphate [IP_3_]-sensitive stores by inhibiting the sarco/endoplasmic reticulum Ca^2+^-ATPase), caffeine (600 nM, an agonist of ryanodine receptors), or dantrolene (600 nM, a ryanodine receptor antagonist) for 4 h prior to examining the intracellular Ca^2+^ levels of SCs. Representative inverted images of BABTA-1 AM fluorescence signals showing the time points of the baseline (F, *left panels of each condition*) and peak (∆F, *light panels of each condition*) levels, and quantification of the relative increase of intracellular Ca^2+^ on SCs are shown. The red circles indicate SCs at baseline (*left)* and peak (*right*) levels. The time interval between the peak and baseline levels is the same in all groups. Graph shows means ± SEM from 10 independent experiments (average of five regions with three transfected cell(s) in the region) Scale bar, 20 µm. ***p = 2.36 × 10^−8^ (ctrl vs. OS); ***p = 7.81 × 10^−5^ (OS vs. mibefradil); *p = 3.60 × 10^−2^ (OS vs. U73122); ***p = 2.86 × 10^−6^ (OS vs. Thaps.); *p = 3.22 × 10^−2^ (Thaps. vs. Thaps. + caffeine); **p = 6.60 × 10^−3^ (Thaps. vs. Thaps. + dantrolene); n.s., non-significant.

We assumed that activation of SC proliferation, differentiation and myelination in the SC monoculture subjected to OS would be accomplished by Ca^2+^ influx into the SC plasma membrane and Ca^2+^ mobilization in the internal Ca^2+^ stores. When constructing the DNA plasmid used for transfection, CatCh exhibited an approximately 70-fold increase in light sensitivity compared to that of wild-type ChR2 following enhanced Ca^2+^ permeability^41^. To examine whether OS induces a change in intracellular Ca^2+^ signals, SCs were treated with various pharmacological Ca^2+^-related interventions. Before examining the intracellular Ca^2+^ level of SCs, we removed extracellular Ca^2+^ and the intracellular Ca^2+^ chelator with BAPTA-1 AM by washing with a standard buffer and regulated extracellular Ca^2+^ during analysis (see Materials and Methods section). We observed a very small difference between the intensity of the resulting peak and the baseline intensity in the Ctrl, Onlystim, and Transf. groups. Indeed, there was no significant difference among the Ctrl, Onlystim, and Transf. groups (Fig. 3a and Suppl. Movie 1). However, when the LED stimulator was applied to transfected SCs, we observed that the Ca^2+^ signal of transfected SCs increased immediately, reaching an intensity 1.7-fold greater than the baseline level. In addition, the ratio of the intensity before versus after OS (ΔF/F) was 3.4-fold higher than that of Transf. (Fig. 3a). Thus, increased intracellular Ca^2+^ may be involved in OS of SCs.

We next examined the pathways through which external Ca^2+^ flows inward across the cell membrane during OS. In SCs, because there are only two types of voltage-gated Ca^2+^ channels (VGCCs)^42–44^, transfected SCs were treated with a T-type VGCC blocker (mibefradil, 40 nM) or an L-type VGCC blocker (nifedipine, 20 nM), which are conventional VGCC blockers used with SCs^43,44^. The ΔF/F value decreased significantly when mibefradil was added for OS of SCs, and the ratio of ΔF/F was reduced by approximately half compared to that of OS without mibefradil (Fig. 3b and Suppl. Movie 1). In contrast, treatment with nifedipine slightly decreased ΔF/F, while there was no statistically significant difference between the treatments with versus without nifedipine during OS of SCs (Fig. 3b and Suppl. Movie 1). These results indicate that external Ca^2+^ influx is mainly generated through the T-type VGCC during OS of SCs.

Two types of internal Ca^2+^ stores, inositol 1,4,5-trisphosphate (IP_3_)-sensitive stores and caffeine/ryanodine-sensitive stores, are reportedly associated with internal Ca^2+^ movement in SCs^45,46^. To examine how Ca^2+^ released within the cell membrane through OS is involved in Ca^2+^ mobilization from these Ca^2+^ stores, we first tested the Ca^2+^ signal from IP_3_-sensitive stores. We used a specific inhibitor of phosphoinositide-specific phospholipase C beta (PLC-β) activation, U73122, to block IP_3_ formation and calcium mobilization from internal IP_3_ stores. After treatment with U73122 (500 nM) during OS of SCs, the peak intensity was significantly inhibited and the ΔF/F value was 0.53-fold attenuated compared to that during OS of untreated SCs (Fig. 3c and Movie 2). Treatment with U73433, which is an inactive analog of U73122, had no effect, and there was no statistically significant difference between treatments with and without U73433 during OS of SCs (Fig. 3c and Suppl. Movie 2). These results indicate that OS of SCs induces an increase in intracellular Ca^2+^ that is mobilized from IP_3_-sensitive stores.

To further examine the participation of Ca^2+^ mobilization from caffeine/ryanodine-sensitive stores, transfected SCs were first treated with thapsigargin (Thaps., 200 nM) to deplete their IP_3_-sensitive stores through inhibition of sarco/endoplasmic reticulum Ca^2+^-ATPase. In OS of SCs treated with Thaps., the ΔF/F value was significantly reduced, whereas treatment with a mixture of Thaps. and caffeine (an agonist of ryanodine receptors; 600 nM) during OS of SCs led to partial recovery of the ΔF/F value (Fig. 3d and Suppl. Movie 2). In contrast, the ΔF/F value decreased drastically when SCs were treated with a mixture of Thaps. and dantrolene (an antagonist of ryanodine receptors; 600 nM) (Fig. 3d and Suppl. Movie 2). These results show that OS of SCs induces increased intracellular Ca^2+^ by promoting flow through the cell membrane via T-type VGCCs, and that this Ca^2+^ is mobilized from both IP_3_-sensitive and caffeine/ryanodine-sensitive stores.

### Promotion of differentiation and myelination of SCs during OS and inhibition through Ca^2+^ blockers in SC-MN coculture

**Figure 4:**
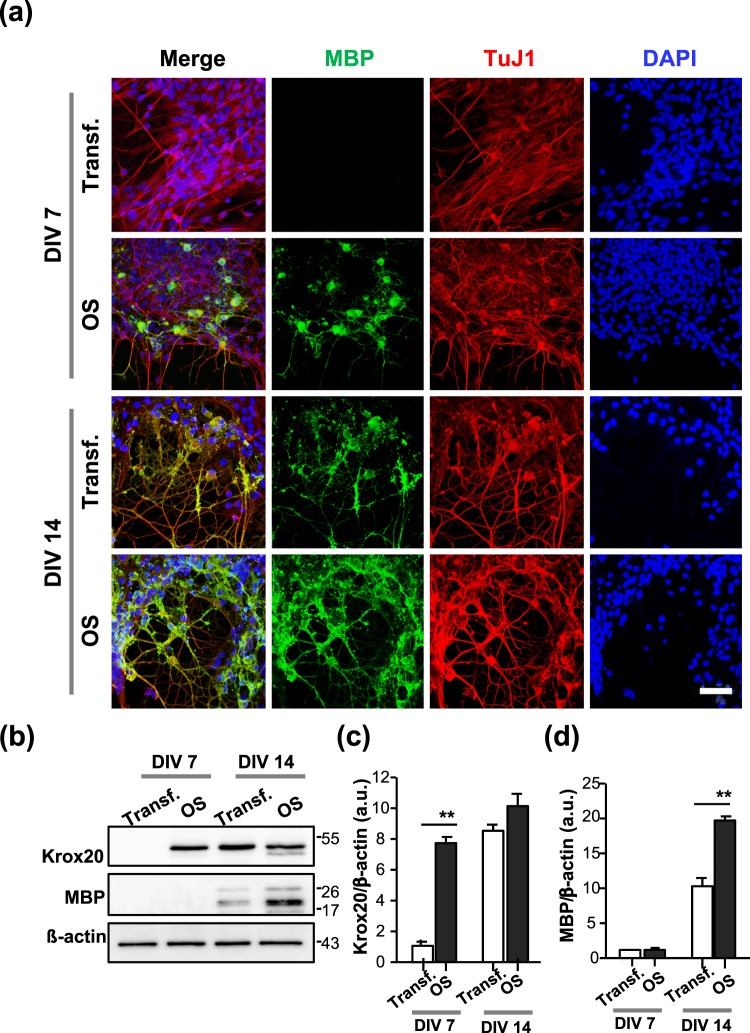
Effect of OS on SC differentiation and myelination in SC-motor neuron (MN) coculture. Transfected SC-MN co-cultures with and without OS were analyzed at DIV 7 and 14 by immunostaining antibodies against MBP (green), tubulin beta III (TuJ1, red), and DAPI (blue). (**a**) Representative confocal images of the SC-MN coculture are shown. Scale bar; 50 µm. (**b**–**d**) The levels of Krox20 or MBP were determined through western blot analysis. (**b**) Representative immunoblots and (**c**,**d**) quantification of Krox20 and MBP protein levels are shown. Note the substantially enhanced expression levels of Krox20 and MBP with OS at DIV 7 and 14, respectively. Protein levels were normalized against the level of ß-actin, which was used as a loading control. Graph shows means ± SEM from five independent experiments (unpaired two-tailed-*t* test with Welch’s correction). **p = 7.78 × 10^−3^ (Krox20); **p = 1.27 × 10^−3^ (MBP).

**Figure 5:**
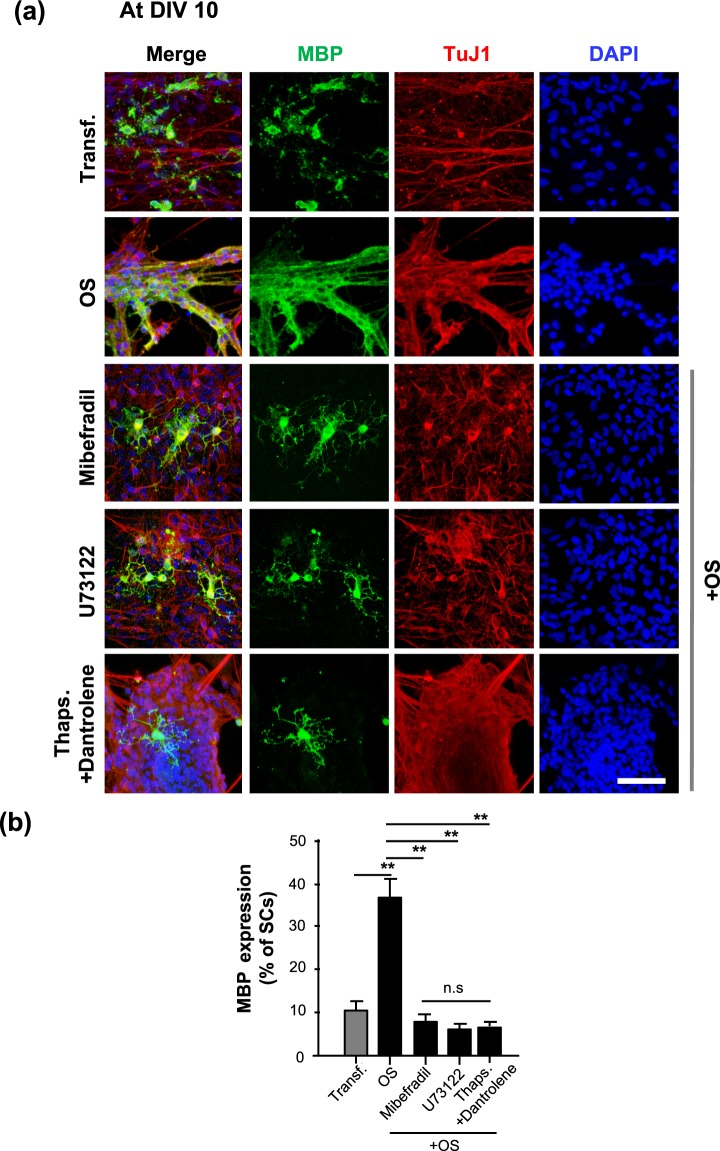
Inhibition of MBP expression in optogenetic-mediated SCs with Ca^2+^ blocker treatment. Transfected SC-MN cocultures were treated with mibefradil, U73122, Thaps. + dantrolene, or no addition at DIV 5, and the concentration of each reagent was maintained throughout the culture until fixation for analysis. The LED irradiator was applied to coculture samples at DIV 7, which were then stimulated for 3 days. (**a**) Representative confocal images of SC-MN coculture stained with MBP, TuJ1, and DAPI at DIV 10 and (**b**) quantification of MBP^+^-SCs are shown. MBP expression was substantially enhanced with OS and, interestingly, the level of MBP protein was inhibited by mibefradil, U73122, or Thaps. + dantrolene. Graph shows means ± SEM from three independent experiments (ANOVA and unpaired two-tailed-*t* test with Welch’s correction). Scale bar, 50 µm. **p = 3.98 × 10^−3^ (Transf.); **p = 3.80 × 10^−3^ (mibefradil); **p = 7.02 × 10^−3^ (U73122); **p = 7.033 × 10^−3^ (dantrolene); **p = 7.02 × 10^−3^ (U73122).

Previous research has shown that MBP expression is initiated at DIV 10 and gradually spreads around the axon at DIV 14 during development of the SC-MN coculture system^33^. To determine whether OS of SCs has an effect on the myelination process in SC-MN coculture, transfected SCs were cocultured with MNs on a coverslip precoated with Matrigel, and then stimulated with or without an LED irradiator. Differentiation and myelination of SCs in the transfected SC-MN cocultures were analyzed at DIV 7 and DIV 14 after the LED irradiator had been applied three times, (i.e., at DIVs 5, 6, and 7). To acquire samples from DIV 7, the SC-MN cocultures were incubated for 8 h after the third LED stimulation and then fixed. Consistent with previous findings, we did not detect MBP expression in the transfected SC-MN coculture without LED stimulation (Transf.) at DIV 7. In contrast, with OS of SCs, MBP expression was initiated when observed at DIV 7, and MBP proteins were growing around the axon (Fig. 4a). Furthermore, the effect of OS on myelination was enhanced at DIV 14. MBP proteins were eventually distributed abundantly throughout the coculture of SCs with OS (Fig. 4a). Western blot analysis confirmed the expression of Krox20 and MBP. With OS of SCs, we observed a strong increase of both Krox20 and MBP protein levels over time (Fig. 4b), consistent with the immunocytochemistry results shown in Fig. 4a. Next, we examined how the increase of MBP protein induced by OS is inhibited by treatment with pharmacological Ca^2+^-related interventions. Before analyzing the expression of MBP protein, we examined whether pharmacological Ca^2+^-related interventions influenced the viability of SC-MN coculture at DIV 10. When treated with each pharmacological Ca^2+^-related intervention in coculture media, nearly the entire SC-MN coculture survived under each condition. These results indicate that each Ca^2+^-related drug does not affect the coculture viability (Suppl. Fig. S2). At DIV 10, we observed that OS-induced MBP protein increase was drastically inhibited by mibefradil, U73122, or a mixture of Thaps. and dantrolene (Fig. 5a). Treatment with mibefradil, U73122, or a mixture of Thaps. and dantrolene during OS of SCs significantly decreased the percentage of MBP-expressing SCs and, importantly, reduced the percentage of MBP-expressing SCs, similar to the Transf. treatment (Fig. 5a,b). These results indicate that OS of SCs promotes the differentiation and myelination of SCs in SC-MN coculture, and that these effects were inhibited by blockade of intracellular Ca^2+^; this suggests that the effect of OS of SCs on the myelination process depends on intracellular Ca^2+^ levels.

## Discussion

Here, we show that OS of SCs not only induces SC proliferation but also promotes differentiation and myelination in SC monoculture and SC-MN coculture, which correlates closely with the intracellular Ca^2+^ level of SCs. In the SC monoculture, SC proliferation was substantially enhanced by OS, and the number of BrdU^+^-S100ß^+^-SCs increased over time. In addition, when dBcAMP/NRG1 was also added to SC culture medium, OS of SCs activated the expression of myelin-associated proteins such as Krox20 and MBP in dedifferentiated SCs, and the redifferentiation and remyelination of SCs was markedly upregulated. These effects of OS depend on the intracellular Ca^2+^ level of SCs, in which the influx of Ca^2+^ through the SC plasma membrane during OS was mainly induced by the T-type VGCC, and Ca^2+^ was mobilized from both IP_3_-sensitive stores and caffeine/ryanodine-sensitive stores. Taken together, these results indicate that the expression of both Krox20 and MBP is also promoted by OS in the SC-MN coculture. Conversely, the expression of these proteins was prevented by the administration of pharmacological interventions related to the Ca^2+^ level, showing that OS may drive changes in diverse SC properties through the regulation of Ca^2+^.

Cell proliferation and differentiation are orchestrated by various proteins related to Ca^2+^ signaling inside the cell. The CatCh that we used is modified with a glial fibrillary acidic protein (GFAP) promoter to activate only glial cells based on the method of Kleinlogel *et al*.^41^ and has higher Ca^2+^ permeability with increasing light sensitivity. Due to the powerful effects of these ChR2 variants on Ca^2+^ contribution, we favor the hypothesis that the potential effect of OS is mainly correlated with Ca^2+^, although other complex signaling mechanisms related to the proliferation and myelination of SCs might be involved. In particular, it is well known that increased intracellular Ca^2+^ in SCs affects their proliferation, differentiation, and myelination^45,47–51^. During the SC proliferation process, the elevation of intracellular Ca^2+^ via a calmodulin (calcium binding protein)-dependent mechanism regulates SC proliferation during the development and regeneration stages. At this point, Ca^2+^ may act as a second messenger for the transduction of mitogenic signals on cultured SCs^47,50^. In the SC myelination process, neuregulin-1 (NRG1), an initiator of myelination, stimulates an increase in cytoplasmic Ca^2+^ and simultaneously binds to erbB2 on SCs. The binding of NRG1 to erbB2 activates PLC-γ, resulting in an elevated intracellular Ca^2+^ level and the activation of calcineurin, which results in the formation of the downstream transcription factor nuclear factor of activated T cells (NFAT) protein complex, NFATc4. The role of the calcineurin/NFAT pathway in Krox20 induction requires an increase in the Ca^2+^ level, which activates the expression of myelin-related genes^52^. Furthermore, the blockade of SC function in differentiation and myelination observed in mice lacking *calcineurin B1*, which inhibits the de-phosphorylation of NFAT proteins, prevents their nuclear entry into NFAT transcription complexes via a Ca^2+^-dependent mechanism^48,49,51^. In particular, PLC-dependent Ca^2+^ influx stimulated by NRG1 leads to differentiation in SC precursors^48,51^. When SCs are cocultured with sensory neurons, the release of intracellular Ca^2+^ in myelinating SCs into Ca^2+^ stores appears to depend upon the progress of myelination, which occurs via ryanodine receptors^45^. In addition, exogenous ATP treatment or electrical stimulation not only induces higher intracellular Ca^2+^ levels in SCs through the P_2y_-purinergic pathway upstream of the SC plasma membrane, but also releases Ca^2+^ into IP_3_- and ryanodine-dependent Ca^2+^ stores; the mitochondrial Ca^2+^ uniporter can promote the myelination process in the presence or absence of axons^45,53,54^. Chronic suppression of Ca^2+^-activated Ca^2+^ release attenuates the development of myelinating SCs^53,54^. The detailed mechanisms of Ca^2+^ signaling remain unknown, but Ca^2+^ is clearly a pivotal regulator of the proliferation and myelination of SCs. Consistent with previous results, we herein demonstrated the contribution of intracellular Ca^2+^ to OS-induced response of SCs, as well as the physiological consequences of SC activation in terms of regulating the proliferation, differentiation, and myelination of SCs.

Using optogenetics, we focused on boosting SC signaling to reveal their contribution to proliferation, differentiation, and myelination processes in the PNS. Functional regulation of SCs is important, as neurons should be supported by SCs for complete formation of the nervous system. Importantly, injured neurons can scarcely survive without SCs; therefore, modulation of the strong effects of SCs on regeneration and remyelination may be necessary to overcome the limitations of current SC therapy. This study offered an exceptional opportunity to elucidate SC functions in proliferation, differentiation, and myelination using optogenetics. Optogenetic SC manipulation may constitute a technical innovation for elucidating the vital role that SCs play in complex higher PNS functions in developmental and neurodegenerative disorders.

## Materials and Methods

### Cell preparation

All acquisition procedures of biological samples were approved by the Institutional Animal Care and Use Committee of the Yonsei University Health System (IACUC of YUHS), and all experiments were conducted in accordance with the relevant guidelines and regulations set by the committee.

#### Schwann cell culture

SC culturing was performed as previously described^33,55^. In brief, sciatic nerves from postnatal day 4 mice were harvested in Ca^2+^- and Mg^2+^-free phosphate-buffered saline (PBS, Lonza) and incubated with 2.5% trypsin (Gibco) and 1 mg/ml collagenase A (Roche) at 37 °C for 30 min. After trypsinization, the pellets were washed with high-glucose Dulbecco’s modified Eagle’s medium (DMEM; Gibco) containing 10% horse serum (HS, Gibco) and then suspended in SC culture medium: high-glucose DMEM with 10% HS, 4 mM L-glutamine (L-gln; Invitrogen), 1% penicillin/streptomycin (P/S; Sigma), 0.5 μM forskolin (Sigma), and 2 ng/ml human heregulin beta-1 (Sigma). After suspension, SCs were cultured on coverslips coated with 10 μg/ml PLL (Sigma). To remove fibroblasts from the SC culture, complement-mediated cytolysis was performed at DIV 2 or 3 as previously described^33^. Briefly, cells were treated with HMEM (DMEM containing 20 mM hydroxyethyl-piperazineethane-sulfonic acid (HEPES) buffer (T&I), 10% HS, 4 mM L-gln, and 1% p/s) for 5 min at room temperature before washing with 20 mM HEPES in Ca^2+^- and Mg^2+^-free Hank’s balanced salt solution (HBSS; Invitrogen). After HMEM solution was removed, 4 ng/ml anti-mouse CD90 (Serotec) in HMEM was added and incubated at 37 °C for 30 min, and 200 µl of rabbit human leucocyte-associated antigens A, B, and C (HLA-ABC)-complemented sera (Millipore) were added and incubated at 37 °C for 1–2 h. Cytolysis was terminated by washing cells with 20 mM HEPES buffer in HBSS.

#### Motor neuron culture

MNs were isolated from the spinal cord of CD-1 mice (embryonic day 12–13 fetuses) as previously described^33,55^. MNs were harvested in Hank’s balanced salt solution (HBSS, Lonza) containing 1% Toll-like receptor (TLR) trypsin (Worthington) for 15 min at 37 °C, followed by treatment with 1% TLR trypsin inhibitor (Sigma). To collect purified MNs, dissociated spinal cord pieces were incubated in an immunopanning culture dish pretreated with p75^NTR^ antibody (Abcam) at room temperature for 1 h. The immunopanning culture dish was washed three times with neurobasal medium (Gibco) containing 1 × Glutamax Ι (Gibco) to remove nerve fragments and p75^NTR^-negative cells. MNs bound to the bottom of panning dish were treated with depolarization solution (0.8% sodium chloride, 30 mM potassium chloride, and 2 mM calcium chloride, Merck) and gently harvested in coculture medium.

#### Coculture of SCs and MNs

The coculture of SCs and MNs was performed as previously described^33,55^. To form the SC feeder layer, SCs cultured on growth factor-reduced Matrigel for 7 days and SCs were transfected with CatCh at DIV 4 in coculture with MNs. MNs were then seeded on top of an SC feeder layer and the SC-MN coculture was grown in coculture medium composed of neurobasal medium, 2% HS, 0.5 mM L-gln, 0.5 μM forskolin, 1% P/S, 1 × B27 supplement (Gibco), 1 mg/ml bovine pituitary extract (Gibco), 10 μg/ml brain-derived neurotrophic factor (BDNF; Gibco), 50 μg/ml ascorbic acid (Sigma), and 1 mM β-mercaptoethanol (Sigma). For the coculture experiment, the SC and MN seeding densities were 2 × 10^4^ and 1.5 × 10^4^ cells/well, respectively, and the SC-MN cocultures were plated onto a 12-well plate.

### DNA construct and cell transfection

pAAV-GFAP-CatCh-EYFP (pCatCh) was constructed and provided by Dr. Eun Mi Hwang of the Center for Functional Connectomes of the Korea Institute of Science and Technology, Seoul, Korea. CatCh, which is ultra-light-sensitive and enhances Ca^2+^ permeability, was used for the transfection of SCs. The GFAP promoter sequence was amplified by PCR from the pAAV-GFAP-EGFP vector (Addgene #50473) using MluI and EcoRI adaptor primers and then cloned into the pAAV-MCS vector (Stratagene). The CatCH-EYFP (L132C) sequence was changed from pAAV-CamKIIa-hChR2(H134R)-EYFP (Addgene #26969) using a mutagenesis kit (Enzynomics), amplified by PCR using EcoRI and BglI adaptor primers and cloned into the pAAV-MCS vector. To transfect SCs, the SCs were treated with a mixture of 5 μg of CatCh and Lipofectamine 2000 reagent (Invitrogen) in Opti-MEM™ medium at DIV 3. After 24 h, SCs were washed three times with PBS and transferred to SC culture medium.

### Measurement of transfection efficiency

Prior to examining the effect of optogenetics on SCs, the efficiency of transfected SCs was tested. Coverslips seeded with SCs (2 × 10^4^ cells) were plated into a 24-well plate. At DIV 7 after CatCh transfection, SC samples were fixed with 4% paraformaldehyde (PFA) and immunostained with anti-green fluorescent protein (GFP) (1:1,000; Abcam) and anti-S100ß (1:300; Abcam), followed by the secondary antibodies Alexa Fluor^®^ 488 donkey anti-rabbit immunoglobulin G (IgG, 1:1,000; Invitrogen) and Alexa Fluor^®^ 568 goat anti-mouse IgG (1:500, Invitrogen). The GFP protein used herein shares approximately 97.8% sequence identity with YFP protein. SC transfection was confirmed by calculating the number of GFP protein-stained SCs. Samples were then counterstained with DAPI (Life Technologies). The number of GFP^+^-S100ß^+^ SCs was calculated from multiple fields of view in confocal fluorescence images and quantified. The total number of S100ß^+^ SCs stained with DAPI was first calculated, and the number of GFP^+^ cells in S100ß^+^ SCs was confirmed (n = 3, five random regions).

### Optical stimulation

To optically stimulate transfected SCs, an LED irradiator was manufactured, similar to those previously described^32^. The LED irradiator was manufactured by assembling a 4 × 6 array of blue LEDs (473 nm wavelength; Eleparts) on a custom-designed printed circuit board (PCB) (Suppl. Fig. S3). LEDs were operated using a power supply (SDG1000 series; Siglent Technologies). To maintain a uniform intensity of LED illumination on transfected SCs, a 45° or 90° lens (Eleparts) was attached to each LED. The intensity of the LED irradiator was measured using an optical power and energy meter (Thorlabs). Transfected SCs were stimulated with an intensity of 5 mW/mm2 at 20 Hz for 1 h. The pattern of optical stimulation consisted of 1 s stimulation/1 s rest with a constant pulse width of 5 ms, according to the protocol optimized by Park *et al*.^32^.

### SC proliferation assay

To examine the effect of optogenetics on SC proliferation, we used the BrdU assay, which measures the incorporation of BrdU, a thymidine analog of DNA. SCs (for the SC proliferation assay, the seeding density was 2 × 10^4^ cells/coverslip plated onto a 24-well plate) were grown for 3 days in SC culture medium containing 10% HS and then transfected with CatCh in SC culture medium containing 5% HS. After 24 h, the SC culture medium was replaced with fresh SC culture medium containing 1% HS and 10 µM of BrdU, and the culture was then incubated at 37 °C for 24 h. The presence of a non-mitogenic concentration of 1% HS was necessary for SC survival. The following day, transfected SCs were either exposed to LED irradiation or not, and fixed with 4% PFA at 0, 1, 3, 6, 12, and 24 h after LED stimulation. As a control, non-transfected SCs were also exposed to LED irradiation or not, and fixed at the times indicated above. Fixed samples were incubated with 1 N HCl on ice and then with 2N HCl at room temperature for DNA hydrolysis. To detect incorporated BrdU, samples were stained with BrdU (1:500; Thermo) and S100β (1:200; Abcam). The number of BrdU^+^-S100ß^+^ SCs was calculated from multiple fields of view under the confocal microscope using ImageJ (n = 5, five random regions).

### Dibutyryl-cAMP and β-neuregulin 1 treatment

To examine the MBP expression level of SCs in the SC monoculture with OS, transfected SCs were stimulated with the LED irradiator or not. SCs seeded onto a coverslip were plated onto a 24-well plate and the plating densities were adjusted (for DIV 1, 3, and 7 samples, the seeding densities were 10 × 10^4^, 7 × 10^4^, and 2 × 10^4^ cells/well, respectively) to obtain a similar number of cells from different DIV samples on the day when the samples were collected. In addition, the MBP expression level of transfected SCs after OS was compared to those in the presence and absence of a mixture of dBcAMP (1 mM; Sigma) and NRG1 (10 nM; Sigma) that induced SC differentiation and myelination in the SC monoculture without neurons. Transfected SCs at DIV 3 were treated with or without the dBcAMP/NRG1 mixture at DIV 4. The following day, transfected SCs were exposed to the LED irradiator at 20 Hz for 1 h, and LED stimulation was applied for 3 days; a parallel culture was not stimulated. Culture samples were fixed at DIV 1, 3, and 7. To harvest samples at DIV 7, SCs were stimulated and, after 8 h, samples were fixed. SC monocultures were stained with S100ß, MBP, and DAPI at the times described above and the number of MBP-expressing SCs was calculated from five different fields of view under the confocal microscope.

### Reagent treatment

To examine how transfected SCs can affect SC proliferation and differentiation through OS, transfected SCs were treated with nifedipine (20 nM; Sigma), U73122 (500 nM; Sigma), U73433 (500 nM; Sigma), caffeine (600 nM; Sigma), mibefradil (40 nM; Abcam), thapsigargin (200 nM; Enzo), or dantrolene (600 nM; Selleckchem), and each reagent was maintained in the culture until analysis of calcium imaging and protein expression levels. In Fig. 5a,b, each reagent was added to SC-MN coculture medium at DIV 5.

### Calcium imaging

To measure the calcium responses of SCs to OS, we used Oregon Green 488 BAPTA-1 AM following the manufacturer’s instructions. Transfected SCs were treated with each reagent at DIV 4 (for calcium imaging, the seeding density was 2 × 10^4^ cells/coverslip). After 24 h, SCs were rinsed three times with standard buffer containing 10 mM HEPES, 4 mM KCl, 2 mM CaCl_2_, 1 mM MgCl_2_, 139 mM NaCl, and 10 mM glucose, followed by treatment with a mixture of 2 mM Oregon Green 488 BAPTA-1 AM and 10% (w/v) pluronic F-127 (at a 1:1 ratio) in standard buffer for 1 h in the dark at room temperature. SCs were washed three times with standard buffer and then subjected to an OS experiment in the presence or absence of each reagent. A single optic fiber was used instead of the LED irradiator (Changchun New Industries Optoelectronics Tech. Co., Ltd.), which was placed near cells under the confocal laser microscope (LSM 700; Zeiss); the intensity of the optical fiber was adjusted to 5 mW/mm^2^, the same intensity as that of the LED irradiator. To examine the intracellular Ca^2+^ level of SCs, the laser power of the confocal microscope was first adjusted to the minimum intensity at which SCs did not respond. Time-lapse images were taken every second with pulse pattern (10 s stimulation/50 s rest) for 5 min at 512 × 512 pixel resolution, and cells were excited at the same intensity using the 488 nm laser line. Under all experimental conditions, the data were excluded after photobleaching was observed during the experiment. Within a given imaging field, only active SCs were chosen for analysis (a minimum of 12–15 cells per condition, n = 10). The intensity of intracellular Ca^2+^ was measured using Zen software, and the intracellular Ca^2+^ level was determined by calculating the ratio between the change in fluorescence signal intensity (∆F) and baseline fluorescence (F).

### Immunocytochemistry

All culture samples were fixed with 4% PFA for 20 min at room temperature, followed by treatment with 0.2% Triton X-100 for 15 min. Samples were soaked with 1% bovine serum albumin (BSA; Millipore) at 4 °C overnight. In Figs 2, 4 and 5, fixed samples were stained with MBP (1:500**;** Abcam), Tuj1 (1:500; Abcam), or S100ß (1:300; Abcam) in 1% BSA (Millipore) at 4 °C overnight, and the secondary antibodies used were goat anti-rat IgG H&L (1:500; Abcam), goat anti-chicken IgY H&L (1:500; Abcam), and goat anti-mouse IgG H&L (1:300; Abcam). Nuclei were stained with DAPI (Life Technologies) for 10 min. All images were acquired using an inverted confocal laser-scanning microscope (LSM 700; Zeiss) equipped with solid-state lasers (405, 488, 555, and 639 nm). Five regions in each condition were chosen at random and analyzed for the number of MBP-positive cells.

### Western blot assays

Expression levels of MBP and Krox20 were quantitatively analyzed using western blot analysis. Samples were lysed in RIPA buffer (T&I) containing 1% SDS and protease inhibitor (aprotinin, leupeptin, pepstatin A, and phenylmethylsulfonyl fluoride). The protein concentration of cell lysates was measured using the Bradford assay (Sigma). The samples were prepared in an SDS sample buffer and heated for 5 min at 95 °C; next, 10 μg of protein from each sample was loaded in SDS loading buffer and transferred to a polyvinylidene fluoride membrane. The membranes were blocked in 5% skim milk for 1 h, followed by incubation with anti-mouse Krox20 (1:1,000; BioLegend) and anti-rat MBP (1:500; Abcam) antibodies at 4 °C overnight. The membranes were washed three times in Tween-20 and incubated with goat anti-mouse IgG or anti-rat IgG conjugated to horseradish peroxidase (1:1,000; Sigma) for 2 h. Bands were visualized using an ECL system. The intensity of the blots was quantified with ImageJ software.

### Measurement of cell viability

To determine cell viability, the LIVE/DEAD cell staining kit (Abcam) was used according to the manufacturer’s instructions. In brief, SC-MN co-cultures were washed once with PBS, followed by labeling with a mixture of solution A (calcein-AM) and solution B (propidium iodide, PI) in PBS at DIV 10. Samples were incubated for 30 min at 37 °C and then rinsed three times with PBS. Samples were examined under a confocal microscope (LSM 700; Zeiss). The numbers of live and dead cells were counted in images acquired from confocal microscopy (n = 3, five random regions).

### Statistical analysis

All statistical analyses were performed with Prism software (GraphPad Software) and the normality of the data was examined using the Shapiro-Wilk test. All statistical data are shown as means ± standard error of the mean (SEM) and comparisons between more than two groups were performed using repeated measurement analysis of variance (ANOVA), while an unpaired two-tailed t-test with Welch’s correction was used to compare the values between two groups. The levels of statistical significance were set to *p* < 0.05, 0.01 and 0.001, and calculated *p*-values are specified in the figure legends.

## Supplementary information

Suppl. Figure 1, 2, 3

Calcium imaging in CatCh-transfected SCs by OS and VGCC.

Calcium imaging in CatCh-transfected SCs by calcium mobilization from internal calcium stores.
