# Supplementary material for: Optogenetic stimulation promotes Schwann cell proliferation, differentiation, and myelination in vitro
Source: Sci Rep. 2019 Mar 5;9:3487. doi: 10.1038/s41598-019-40173-w (PMC6401157; doi:10.1038/s41598-019-40173-w)
Supplement: Supplementary file 1 — Suppl. Figure 1, 2, 3 [file 41598_2019_40173_MOESM1_ESM.pdf]

Optogenetic stimulation promotes Schwann cell proliferation, differentiation, and myelination *in vitro*

Kyuhwan Jung[1†], Ji Hye Park[2], Sung-Yon Kim[3], Noo Li Jeon[4,5*], Sung-Rae Cho[1,6*] & Sujin Hyung[4,7,8 †*]

[1] Graduate Program of Nano Science and Technology, Graduate School of Yonsei University, Seoul, Korea

[2] Gradaute Program of Translational Neuroscience, Institute for Clinical Neurobiology, University of Wuerzburg, Germany

[3] Department of Biophysics and Chemical Biology, Seoul National University, Seoul, South Korea

[4] Multiscale Mechanical Design School of Mechanical and Aerospace Engineering Institute of Advanced Machinery and Design, Seoul National University, Seoul, Korea

[5] Institute of Bioengineering, Seoul National University, Seoul, Korea

[6] Department and Research Institute of Rehabilitation Medicine, Yonsei University College of Medicine, Seoul, Korea

[7] BK21 Plus Transformative Training Program for Creative Mechanical and Aerospace Engineers, Seoul National University, Seoul, Korea

[8] present address: Department of Molecular Biology and Genetics, Weill Institute for Cell and Molecular Biology, Cornell University, Ithaca, USA

[†]Both authors contributed equally to this work

*Co-Corresponding Authors: **Sujin Hyung, Ph.D.**

E-Mail: smsns7230@gmail.com

Sung-Rae Cho, M.D., Ph.D.

E-Mail: SRCHO918@yuhs.ac

Noo Li Jeon, Ph.D.

E-Mail: njeon@snu.ac.kr

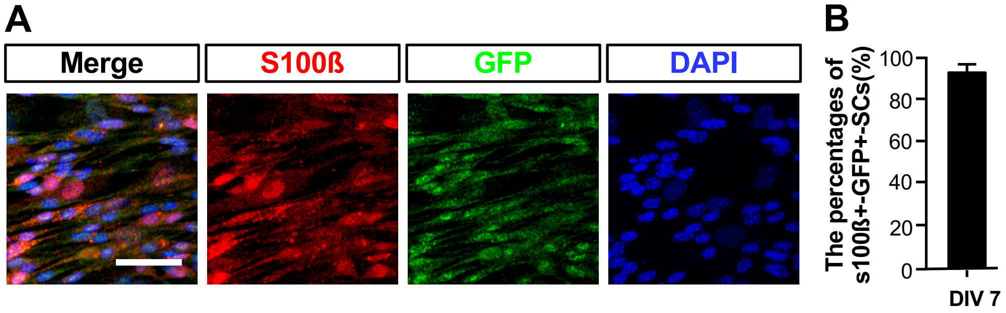

Suppl. Fig. 1. Transfection efficiency of CatCh-transfected SCs in the SC monoculture. Cultured SCs were transfected with 5 µg of CatCh at DIV 3 and stained with green fluorescent protein (GFP) (green), S100ß (red), and DAPI (blue) at DIV 6. CatCh transfection of SCs was confirmed using GFP imaging. Representative confocal images (**A**) of SCs and (**B**) quantification of GFP^{+}-$S100ß^{+}$-SCs are shown. The transfection efficiency, as evaluated from GFP^{+}-$S100ß^{+}$ co-labeling, was 92.4 ± 2.6%. The graph shows means ± SEM from three independent experiments. Scale bar, 50 µm.

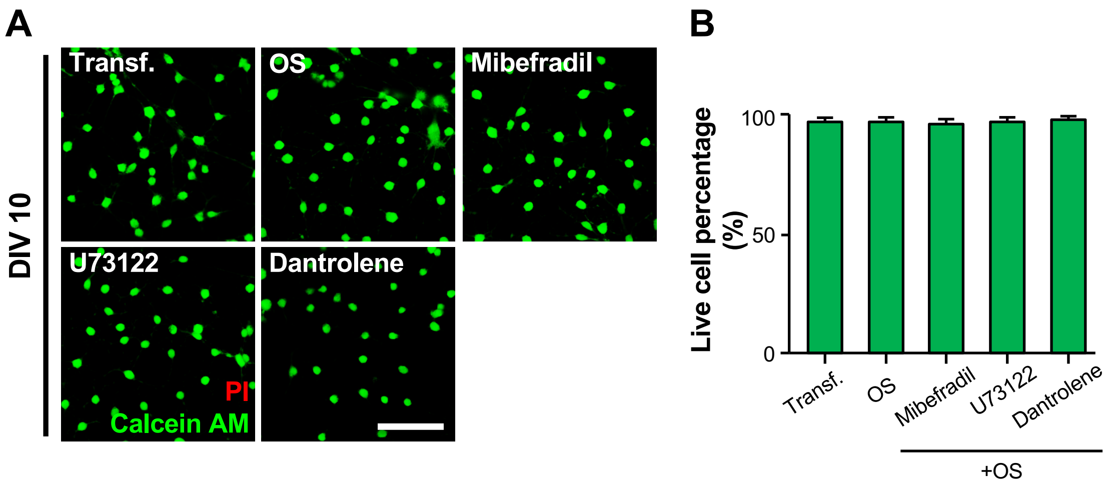

Suppl. Fig. 2. Cell viability in the SC-MN coculture. (**A** and **B**) Viability of the SC-MN coculture was determined using calcein-AM (green)/ propidium iodide (PI; red) double staining at DIV 10. (**A**) Representative images and (**B**) quantification of live cells are shown. Cells in the SC-MN coculture almost all survived and there were no significant differences among groups. The graph shows means ± SEM from three independent experiments. Scale bar, 50 μm.

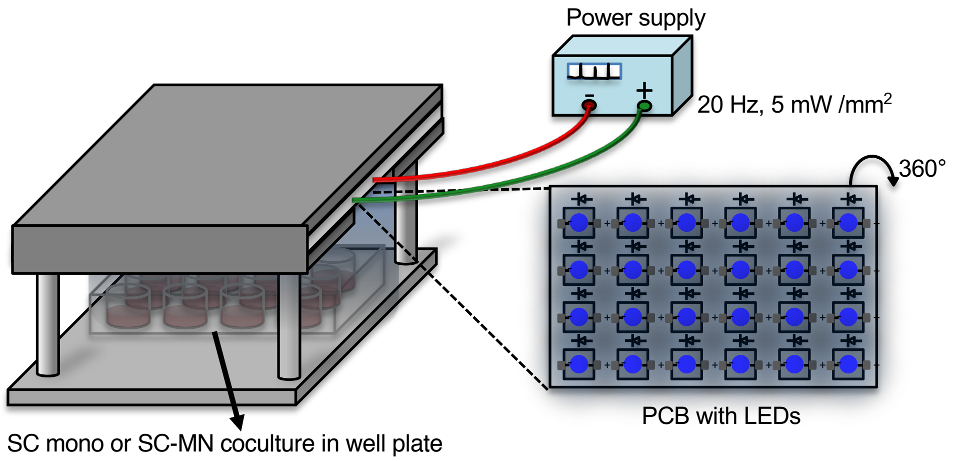

Suppl. Fig. 3. The design of a custom LED irradiator for OS. A schematic of the LED irradiator that was manufactured by assembling a 4 × 6 array of blue LEDs (473 nm wavelength) on a custom-designed printed circuit board (PCB). The LED irradiator was applied to SCs or SC-MN cocultures at 20 Hz for 1 hr.

Suppl. Movie 1. Calcium imaging in CatCh-transfected SCs by OS and VGCC. This video shows optogenetic-mediated Ca^{2+} transients in transfected SCs labeled with BAPTA-1 AM when SCs were exposure OS and treated with VGCC blockers. Scale bar, 50 μm.

Suppl. Movie 2. Calcium imaging in CatCh-transfected SCs by calcium mobilization from internal calcium stores. This video shows optogenetic-mediated Ca^{2+} transients in transfected SCs labeled with BAPTA-1 AM when SCs were treated with inhibitors of the activity of the IP_3-sensitive or caffeine/ryanodine-sensitive stores. Scale bar, 50 μm.
